# Association between copper intake and essential hypertension: dual evidence from Mendelian randomization analysis and the NHANES database

**DOI:** 10.3389/fnut.2024.1454669

**Published:** 2024-08-29

**Authors:** Qing Miao, Jingtao Zhang, Yingjie Yun, Wei Wu, Chuanjin Luo

**Affiliations:** ^1^The First Clinical Medical College of Guangzhou University of Chinese Medicine, Guangzhou, China; ^2^The First Affiliated Hospital of Guangzhou University of Chinese Medicine, Guangzhou, China

**Keywords:** copper intake, essential hypertension, Mendelian Randomization, National Health and Nutritional Examination Survey, trace elements

## Abstract

**Background:**

Although previous studies have identified an association between trace elements and essential hypertension, the specific trace elements involved and the mechanisms of their association remain unclear. This study aimed to elucidate the relationship between various human trace elements and essential hypertension, thereby addressing existing gaps in the research.

**Methods:**

This study employed two-sample, multivariate, and inverse Mendelian randomization (MR) analyses to investigate the causal relationship between 15 human trace elements as exposure factors and essential hypertension as the outcome. The analysis revealed a statistically significant association between copper intake and essential hypertension. Further validation was conducted using logistic regression models based on data from the National Health and Nutrition Examination Survey (NHANES).

**Results:**

Eighteen trace elements were initially identified through searches in the GWAS database and PubMed. After screening, 15 trace elements were selected as potential exposure factors. MR analysis, utilizing the 2021 genome-wide dataset for essential hypertension, identified copper as a risk factor, showing a positive association with hypertension. Subsequent logistic regression analyses based on NHANES data further confirmed a significant association between dietary copper intake and the risk of essential hypertension, except for the 0.80–1.08 mg/d group in model 3 (*p* < 0.05). Restricted cubic spline (RCS) analysis indicated a nonlinear relationship between copper intake and the risk of developing essential hypertension.

**Conclusion:**

This study demonstrates a significant association between copper intake and the development of essential hypertension. The findings suggest that higher copper intake is linked to an increased risk of hypertension, underscoring the need to monitor copper intake levels in the prevention and management of this condition.

## 1 Introduction

Essential hypertension is a cardiovascular disease characterized by elevated arterial pressure within the systemic circulation. Persistent hypertension can lead to significant damage to target organs, including blood vessels, the heart, brain, kidneys, and retina, ultimately resulting in severe cardiovascular and cerebrovascular events such as congestive heart failure, stroke, and chronic renal failure. It is a major contributor to mortality from cardiovascular and cerebrovascular diseases (1). Recent surveys indicate that the global prevalence of hypertension is rising steadily, with an increasingly younger age of onset, posing a significant societal burden (2–4). Despite extensive research, the etiology and pathogenesis of hypertension, particularly essential hypertension, remain incompletely understood (5, 6). Consequently, identifying the pathogenic factors underlying essential hypertension is crucial for its prevention and treatment.

Trace elements, although present in the human body in minute quantities (typically less than 0.01% of body weight) are vital for maintaining normal physiological functions and overall health. These elements play essential roles in enzyme catalysis, hormone synthesis and secretion, antioxidant defense, DNA synthesis and repair, and cell signaling (7, 8). Recent studies have increasingly linked trace elements to the development of various diseases, including essential hypertension. For instance, selenium, a key antioxidant, is involved in protecting the cardiovascular system from oxidative stress and may contribute to cardiovascular disease prevention (9). Zinc is implicated in blood pressure regulation through its effects on immune function and angiotensin-converting enzyme activity (10, 11). Organic germanium has been observed to exert a sustained hypotensive effect, effectively reducing both systolic and diastolic blood pressure and alleviating hypertension symptoms (12). Copper, similarly, has been associated with blood pressure regulation, influencing it through its role in norepinephrine synthesis (13, 14). Several other trace elements have also been found to have associations with essential hypertension (15, 16).

The Mendelian randomization (MR) method is a powerful tool for evaluating potential causal relationships between risk factors and diseases. This approach uses genetic variation as an instrumental variable to assess the impact of specific risk factors. Since genotypes are randomly assigned at conception, the observed genetic variations are free from confounding factors such as environmental exposures and are unaffected by disease onset. In this study, a two-sample, multivariate, inverse Mendelian randomization analysis was conducted using 15 micronutrients as exposures and essential hypertension as the outcome. The findings revealed a positive association between copper, one of the micronutrients, and essential hypertension. To further investigate this relationship, we conducted a detailed clinical data analysis using the National Health and Nutrition Examination Survey (NHANES) database, aiming to clarify both the quantitative and qualitative relationships between dietary copper intake and essential hypertension.

## 2 Materials and methods

### 2.1 Data sources for Mendelian randomization

#### 2.1.1 Genetic epidemiologic data on micronutrients

This study searched the Genome-Wide Association Studies (GWAS) catalog^1^ and Pubmed^2^ (last checked April 2024) for published data on mineral and vitamin circulating concentrations in published GWAS data. A total of 18 trace minerals were retrieved, including Copper, Calcium, Carotene, Folate, Iron, Magnesium, Potassium, Selenium, Retinol, Vitamin A, Vitamin B1, Vitamin B2, Vitamin B6, Vitamin B12, Vitamin C, Vitamin D, Vitamin E, and Zinc. of these, Vitamin B1 and Vitamin B2 were excluded because genome-wide significant results were not reported or GWAS studies were not performed. Retinol was also excluded because its data were adjusted for body mass index (BMI) which could lead to biased Mendelian randomization (MR) estimates. GWAS data for 15 micronutrients were finally screened for Copper (ieu-a-1073), Calcium (ukb-b-8951), Carotene (ukb-b-16202), Folate (ukb-b-11349), and Iron (ukb-b-20447), Magnesium (ukb-b-7372), Potassium (ukb-b-17881), Selenium (ieu-a-1077), Vitamin A (ukb-b-9596), Vitamin B12 (ukb-b-19524), Vitamin B6 (ukb-b- 7864), Vitamin C (ukb-b-19390), Vitamin D (ukb-b-18593), Vitamin E (ukb-b-6888), and Zinc (ieu-a-1079). These data were used as exposure factors in a subsequent two-sample Mendelian randomization study.

#### 2.1.2 Genetic epidemiologic data on essential hypertension

We searched the FinnGen Research Program (FINNGEN)^3^ database for genetic epidemiology data on essential hypertension. Considering sample size, sequencing depth, ethnicity, and data update time, we selected a genome-wide genetic dataset on essential hypertension uploaded in 2023^4^. This dataset included 102,864 cases and 289,117 control individuals, totaling 16,380,466 SNPs. All studies included in FINNGEN were approved by the relevant ethical review boards and participants provided informed consent. The current study used only publicly available summary-level data and therefore did not require additional ethical review.

### 2.2 Mendelian randomization analysis

#### 2.2.1 Removal of weak instrumental variables

To satisfy the association assumption, SNPs had to be strongly correlated with exposure factors, and to ensure independence among SNPs and to remove result bias due to chain imbalance. In this study, SNP data were screened by R software, and the filtering criteria were (1) SNPs included in the instrumental variables were correlated with exposure factors (*p* < 1 × 10^−5^), and due to the fact that the number of SNPs that could reach the genome-wide significance level (*p* < 5 × 10^−8^) of the SNPs are fewer in number, a single or very small number of SNPs may not capture enough genetic variation, so using a looser *p*-value threshold increases the number of SNPs available for MR analyses and enhances the overall instrumental variable strength; (2) exclude SNPs that have an *R*^2^ > 0.001 with the most significant SNP in the 10,000 kb range, with *R*^2^ being the proportion of the variability in the risk factors explained by the SNPs, *R*^2^ = 2 × (1 − MAF) × MAF × (*β*₁/SD)^2^, where MAF is the minor allele frequency of exposure, *β*₁ is the allele effect value of exposure, and SD is the standard deviation; (3) SNPs with an *F*-statistic >10, *F* = (*N* − 2) × *R*^2^/(1 − *R*^2^), and *N* is the sample size to obtain SNPs that were strongly correlated with the exposure factors and were independent of each other as effective Instrumental variables. To ensure that each instrumental variable was associated with the same effector allele, this study harmonized the summary statistics and eliminated palindromic SNPs (17).

#### 2.2.2 Two-sample Mendelian randomization analysis

Instrumental variable data for the outcome were obtained using R, and effect sizes were combined. The exposure and outcome data were then preprocessed to ensure consistent formatting. Subsequently, two-sample Mendelian randomization (MR) analyses were conducted to assess the causal relationship between the exposure factor and the outcome variable, using odds ratios (OR) as the measure of association. The inverse variance weighted (IVW) method, a commonly used statistical approach in two-sample MR analysis, was employed. This method is characterized by excluding the intercept term in the regression and using the inverse of the outcome variance as the weighting factor. The reliability of IVW results depends on the absence of heterogeneity and horizontal pleiotropy in the instrumental variables (18). Additionally, the MR-Egger method was utilized, as it can provide valid estimates even in the presence of horizontal pleiotropy among instrumental variables. The weighted median method was also applied, offering the advantage of producing reliable MR estimates as long as more than half of the instrumental variables are valid. This method allows for the inference of causal relationships based on the majority of valid instrumental variables (19). When heterogeneity is present without horizontal pleiotropy, the weighted model approach is preferred, and the IVW random effects model can be employed. Conversely, the simple model approach can be used as a methodological complement to assess the robustness of MR analysis results.

#### 2.2.3 Sensitivity analysis

To ensure the reliability of the causal effect assessment, sensitivity analyses were conducted. The robustness of the causal findings was evaluated using the leave-one-out method, which tests the influence of each instrumental variable on the overall results. If excluding a particular instrumental variable significantly alters the overall results, it suggests that this variable may be a key component in the MR analysis or that there may be issues such as genetic bias. Heterogeneity tests were also performed as part of the two-sample MR analyses to determine whether there were differences among the instrumental variables. Cochran’s *Q* test was employed to assess heterogeneity; a *p*-value greater than 0.05 indicates the absence of heterogeneity, whereas a *p*-value less than 0.05 suggests its presence. Furthermore, the presence of horizontal pleiotropy was evaluated, which occurs when instrumental variables directly affect the outcome variables independently of the exposure factor, thereby violating the exclusivity assumption of MR. If horizontal pleiotropy is detected in the MR analysis results, it indicates that the findings may not be reliable (20).

#### 2.2.4 Multivariate Mendelian randomization analysis

As an extension of two-sample MR, multivariate MR can be employed to estimate the collective causal effects of multiple risk factors on essential hypertension. By incorporating all exposures into a single model, multivariate MR allows for the assessment of the independent direct effects of individual micronutrients on essential hypertension, free from mediation by other exposure factors. In this study, we identified SNPs that were significantly associated with the exposures of interest and combined them with existing instrumental variables. After removing duplicate SNPs, we calculated the effect size and corresponding standard error for each SNP from both the exposure and outcome datasets. The inverse variance weighted (IVW) method, based on weighted linear regression, was then applied to infer causality in the multivariate MR analyses (21).

#### 2.2.5 Reverse Mendelian randomization analysis

**Figure 1:**
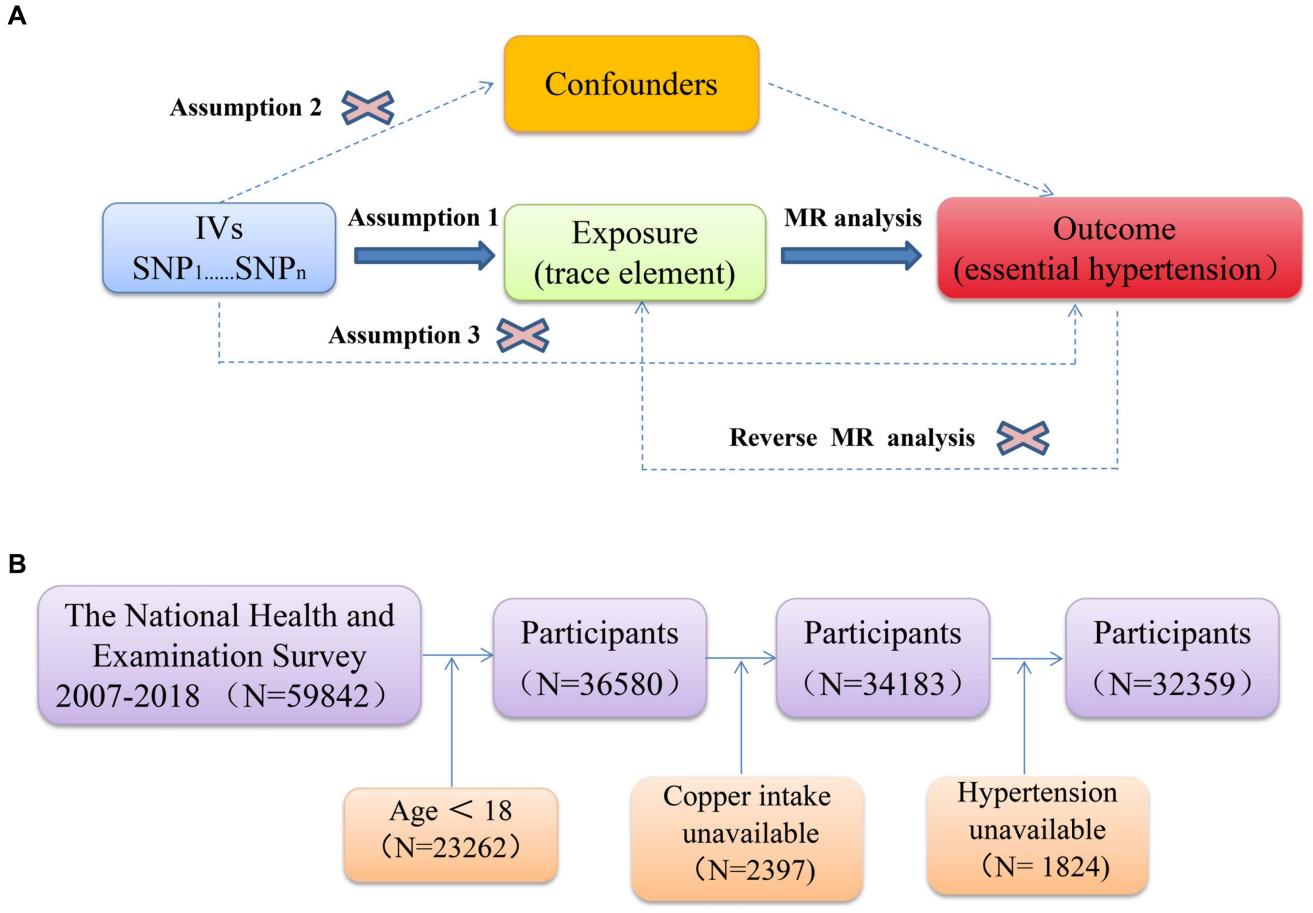
**(A)** Schematic of Mendelian randomization; **(B)** flowchart depicting the participants’ selection.

Reverse Mendelian randomization is a method used to assess causal relationships by treating disease states as exposure variables and potential risk factors as outcome variables. This approach is particularly effective in investigating scenarios where reverse causality may be present—situations in which the disease could influence the exposure factor. In this study, essential hypertension was used as the exposure variable, while the trace elements identified through Mendelian randomization, as previously described, served as the outcome variables. A reverse Mendelian randomization analysis was then conducted to explore and confirm the potential reverse causality between trace elements and essential hypertension. Figure 1A illustrates the schematic diagram and workflow of the Mendelian randomization study.

### 2.3 Analysis of the NHANES database

#### 2.3.1 Sources of information and study populations

The National Health and Nutrition Examination Survey (NHANES) is an ongoing, large-scale, representative, national survey of health and nutrition status conducted by the National Center for Health Statistics (NCHS), a division of the Centers for Disease Control and Prevention (CDC) (22). The protocol for NHANES was approved by the NCHS Research Ethics Review Board (23), and informed written consent was obtained from all participants; therefore, ethical approval for this study was waived. A total of 59,842 participants were surveyed in this cross-sectional study that included data from six cycles from 2007–2018, and the detailed NHANES study design and data are publicly available on the^5^ website.

#### 2.3.2 Definitions of copper intake

Data on dietary copper were extracted from the “Dietary Interview—Total Nutrient Intakes, First Day,” “Dietary Interview—Total Nutrient Intakes, Second Day,” and “Dietary Supplement Use 30-Day—Total Dietary Supplements” datasets within the Dietary Data module. To ensure data completeness, the average dietary copper intake from Day 1 and Day 2 was calculated and combined with the amount of dietary copper from supplements to obtain the total dietary copper intake. This approach assumed that data were not missing for either day. If copper intake data were available only for Day 1 or Day 2, the intake for that day was combined with the dietary supplement data to estimate total copper intake. Following the Joint FAO/WHO Expert Committee on Food Additives (JECFA) guidelines, which set the upper limit (UL) of copper intake for adults at 10 mg/day, any data indicating a copper intake greater than 20 mg/day were excluded to avoid the influence of extreme values.

#### 2.3.3 Essential hypertension ascertainment

The diagnosis of essential hypertension requires a systolic blood pressure ≥140 mmHg and/or a diastolic blood pressure ≥90 mmHg, measured on three separate occasions on the same day, without the use of antihypertensive medication. Blood pressure data from the NHANES database were obtained from three consecutive measurements taken on the same day. To identify individuals with hypertension, we utilized responses to the question “BPQ 020” (“Have you ever been told you have high blood pressure?”), as previous studies have demonstrated a strong correlation between self-reported hypertension and clinical confirmation. Additional health conditions were identified using relevant questions from the NHANES database (24). For instance, sleep apnea syndrome was diagnosed using the question “How often do you snort/stop breathing?” Thyroid disease was identified through the question “Do you still have a thyroid problem?” and kidney disease was diagnosed with “Ever told you had weak/failing kidneys?” Family history of hypertension or stroke was determined using the question “Blood relatives w/hypertension/stroke. “Heart valve disease was diagnosed based on the question “Had heart valve problem?” and chronic obstructive pulmonary disease (COPD) was identified with the question “Ever told you had COPD?” Pregnancy-related hypertension was assessed using the pregnancy test results in the laboratory panel, and current use of painkillers, hormones, and contraceptives was determined by questions such as “Ever taken prednisone or cortisone daily?,” “Drugs injected - Steroids,” and “Taking estrogen/progestin now?” Individuals with a diagnosis suggestive of secondary hypertension were excluded, resulting in a final cohort of participants with essential hypertension.

#### 2.3.4 Assessment of covariates

The study collected data on various demographic, socioeconomic, and health-related variables, including age, gender, ethnicity, education level, marital status, household poverty-to-income ratio, smoking status, alcohol consumption, physical activity, and disease status (e.g., diabetes, cardiovascular health, high cholesterol levels, sleep disorders, and mental health). This information was gathered through standardized questionnaires. Additionally, participants’ body weight and body mass index (BMI, kg/m^2^) were measured at a mobile health check-up center and included as covariates in the analysis.

Age, weight, BMI, and household poverty-to-income ratio were treated as continuous variables, while ethnicity, education level, marital status, smoking status, alcohol consumption, physical activity, and disease status (e. g. diabetes, cardiovascular health, high cholesterol levels, and sleep disorders) were categorized as categorical variables. Ethnicity was classified as Mexican American, other Hispanic, non-Hispanic White, non-Hispanic Black, or other races. Education level was categorized into less than high school, high school or equivalent, and college or above. Alcohol consumption was defined as consuming ≥4 drinks per day, and smoking status was determined by whether participants had smoked more than 100 cigarettes in their lifetime. Marital status was classified as living with a partner or living alone, and physical activity was assessed by asking, “In a typical week, do you do any moderate-intensity exercise?” Self-reported disease status, including cardiovascular and other conditions, was obtained through personal interviews using standardized medical condition questionnaires, such as “Have you ever been told you have a sleep disorder?,” “Have you ever been told you have diabetes?,” and “Have you ever been told you have high cholesterol levels?” Participants answered these questions with “yes” or “no.” Previous research has shown a good correlation between self-reported conditions and clinical diagnoses (25). Further details regarding the covariates are available on the NHANES website.

#### 2.3.5 Statistical analysis

Data from this study were analyzed following CDC guidelines. Participant characteristics were presented as means with 95% confidence intervals (CIs) for continuous variables and as counts with percentages for categorical variables. Baseline characteristics between groups were compared using analysis of variance (ANOVA) for normally distributed continuous data, the chi-square test for categorical variables, and the Kruskal–Wallis H test for non-normally distributed continuous data. The U. S. Institute of Medicine (IOM) of the National Academy of Sciences provides a Recommended Dietary Allowance (RDA) for copper, which is 0.9 mg/day for adults (26). Based on quartiles of dietary copper intake, the study population was divided into five groups: <0.80 mg/d, 0.80–1.08 mg/d, 1.08–1.45 mg/d, 1.45–2.29 mg/d, and >2.29 mg/d. Baseline characteristics were plotted accordingly. To enhance data visualization, forest plots were created for dichotomous variables, using a dietary copper intake of 0.8 mg/d as the cut-off, and subgroup analyses were conducted for covariates.

Logistic regression was then employed to examine the association between dietary copper intake and the development of hypertensive disorders, adjusting for potential confounders. Odds ratios (ORs) and their 95%CIs were calculated to assess the relationship between dietary copper intake and hypertensive outcomes, with four models being constructed: Model 1 was unadjusted; Model 2 adjusted for age, sex, race, education level, and marital status; Model 3 further adjusted for household poverty-to-income ratio (PIR), alcohol intake, smoking status, body mass index (BMI), and physical activity; and Model 4 additionally adjusted for hypercholesterolemia, diabetes cardiovascular disease, and sleep disorders. The group with the lowest copper intake served as the reference group in all models.

Finally, the non-linear relationship between copper intake and essential hypertension was analyzed using a restricted cubic spline (RCS) with three knots at the 10th, 50th, and 90th percentiles. All statistical analyses were performed using SPSS Statistics version 26 and R version 4.3.2. Statistical significance was determined by a two-sided test, with *p* < 0.05 indicating a statistically significant difference. Figure 1B illustrates the study flow for the NHANES analysis.

## 3 Results

### 3.1 Instrumental variable filtering results

**Figure 2:**
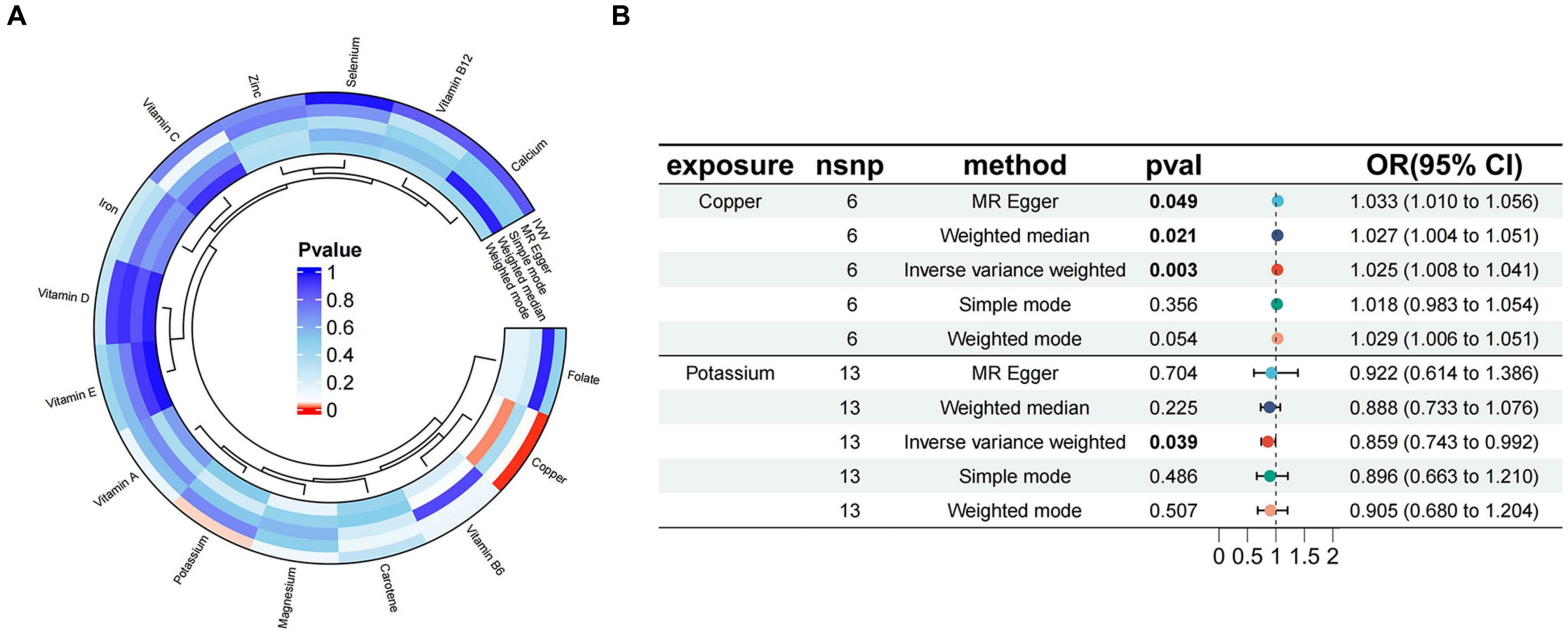
**(A)** Circle plot visualizing the results of Mendelian randomization between 15 micronutrients and essential hypertension; **(B)** Mendelian randomized forest plot of copper, potassium, and essential hypertension.

The results of Mendelian randomization between 15 trace elements and essential hypertension were visualized based on the IVW method, as shown in Figure 2A. Two micronutrients were screened out from the 15 micronutrients that were associated with essential hypertension (*p* < 0.05), Copper (6 SNPs) and Potassium (13 SNPs). A total of 181 SNPs were included in the analyses, all of which had *F* values >10.

### 3.2 Results of the MR analysis

The results of MR analysis showed that Copper and Potassium were significantly associated with essential hypertension. The results of the analyses for Copper were MR-Egger *β* = 0.032, *p* = 0.049; IVW *β* = 0.024, *p* = 0.003, indicating that when Copper was used as an exposure factor, the composite effect value *β* was greater than 0, suggesting that it is a risk factor for essential hypertension, and the risk of developing the outcome increased with increased exposure. Potassium the results of the analyses were MR-Egger *β* = −0.081, *p* = 0.704; IVW *β* = −0.152, *p* = 0.038, indicating that when Potassium was used as an exposure factor, the composite effect values *β* were all less than 0, suggesting that it is a protective factor and that the risk of developing the outcome decreases with increasing exposure. Copper and Potassium and Essential Hypertension the Mendelian randomised forest plot of Copper and Potassium is shown in Figure 2B.

### 3.3 Effect of exposure factors on outcome variables

**Figure 3:**
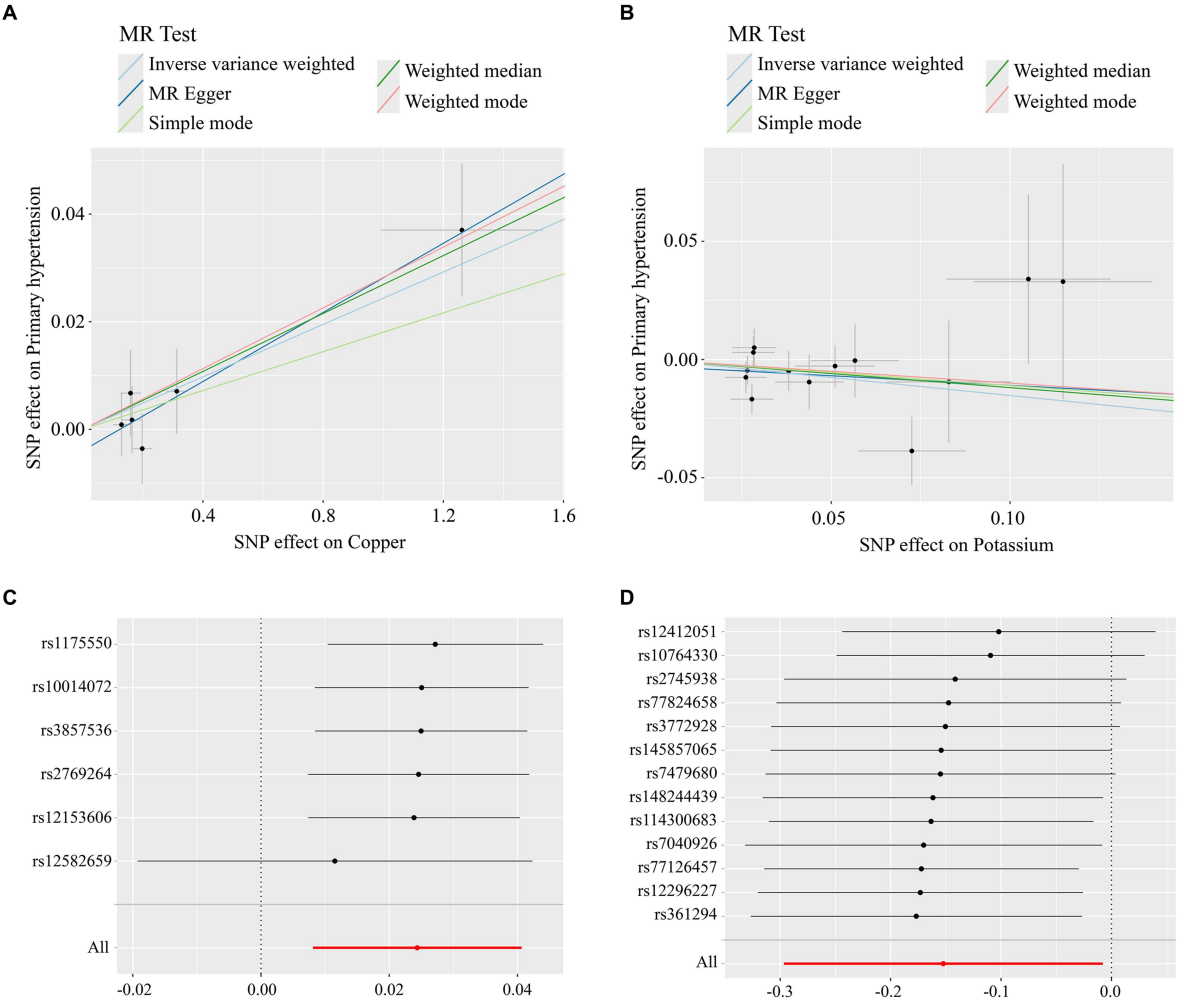
**(A)** Scatterplot of Mendelian randomisation results for copper; **(B)** scatterplot of Mendelian randomisation results for trace element Potassium; **(C)** Mendelian randomisation of leave-one-out graphs for elemental copper; **(D)** Mendelian randomisation of leave-one-out graphs for elemental potassium.

The MR analysis results of the 2 micronutrients that showed a causal effect of exposure and outcome were visualized concerning the MR analysis results of essential hypertension. As shown by the scatter plot (Figures 3A,B), the black dots were distributed in a concentrated manner, suggesting a causal relationship between exposure and outcome, and the overall direction of the lines obtained by different methods was consistent. The slopes of the lines corresponding to Copper were all >0, indicating a positive correlation between exposure and outcome, while the slopes of the lines corresponding to Potassium were all <0, indicating a negative correlation between exposure and outcome. Based on the IVW method, the OR of Copper [OR = 1.024, 95%CI (1.008, 1.041), *p* = 0.003] was greater than 1, indicating that it was a risk factor, and the OR of Potassium [OR = 0.085, 95%CI (0.743, 0.092), *p* = 0.039] was less than 1, indicating that it was a protective factor.

### 3.4 Results of sensitivity analysis

#### 3.4.1 Heterogeneity test

Instrumental variables were tested for heterogeneity using MR-Egger and IVW methods. Copper (MR-Egger *Q* = 1.349, *p* = 0.852; IVW *Q* = 2.305, *p* = 0.805) and Potassium (MR-Egger *Q* = 13.924, *p* = 0.237; IVW *Q* = 14.096, *p* = 0.294) had *p*-values >0.05, indicating that there was no heterogeneity in the SNPs of the instrumental variables.

#### 3.4.2 Horizontal multiple validity test

Horizontal multiple validity test of instrumental variables using both MR-Egger intercept test and MR-PRESSO global test showed that Copper (intercept tended to be 0; RSSobs = 0.631, *p* = 0.704), Potassium (intercept tended to be 0; RSSobs = 16.760, *p* = 0.291), and the results obtained by The intercept terms obtained by MR-Egger intercept test converged to 0 and *p*-value >0.05, while the *p*-values obtained by the MR-PRESSO global test were all >0.05, suggesting that there is no horizontal multiplicity in the instrumental variables of the exposure factors.

#### 3.4.3 Leave-one-out test

When any one SNP was removed and the MR analysis was repeated, no significant difference in overall causality was found, indicating that the results were not due to a single SNP. As shown in Figures 3C,D.

### 3.5 Multivariate Mendelian randomisation

Multivariate MR analyses were performed separately to correct for the interaction between various micronutrients and essential hypertension. The results showed that Potassium was not a protective factor for essential hypertension (OR = 0.798, 95%CI: 0.067–1.049, *p* = 0.105), while Copper remained a risk factor for essential hypertension (OR = 1.025, 95%CI:1.008–1.449, *p* = 0.003).

### 3.6 Reverse MR analysis

The results for MR Egger, Weighted Median Estimator, IVW, Simple Mode, and Weighted Mode were (OR = 1.090, 95%CI: 0.911–1.303, *p* = 0.416), (OR = 1.018, 95%CI:0.990–1.046, *p* = 0.219), (OR = 1.006, 95%CI: 0.971–1.042, *p* = 0.757), (OR = 1.025, 95%CI: 0.992–1.060, *p* = 0.215), (OR = 1.020, 95%CI: 0.990–1.052, *p* = 0.264). The *p*-values of the above 5 research results are all greater than 0.05, the results suggest that essential hypertension is not a causal factor for copper intake and there is no significant causal relationship between the two.

### 3.7 Baseline characteristics of study participants

After the exclusion of age less than 18 years, invalid dietary copper intake data, and hypertension data, 32,359 subjects were finally included in this study. Five observation groups (<0.8 mg/d, 0.80–1.08 mg/d, 1.08–1.45 mg/d, 1.45–2.29 mg/d, >2.29 mg/d) were divided according to dietary copper intake and a table of baseline characteristics of the population was produced (Table 1). The results showed a higher proportion of females in the lower copper intake group compared to the normal copper intake group (*p* < 0.001), with a decreasing proportion of the female population as copper intake increased; the lower copper intake group also had a higher proportion of sleep disorders (*p* < 0.001). There was no statistically significant difference in alcohol consumption levels between groups (*p* = 0.292).

**Table 1:** Baseline characteristics of patients with essential hypertension according to the amount of copper intake.

Characteristics	Copper intake (mg/d) (32359)					*p*
	<0.80	0.80–1.08	1.08–1.45	1.45–2.29	>2.29	
	*N* = 7,934	*N* = 8,143	*N* = 8,119	*N* = 6,556	*N* = 1,607	
**Age (years)**	48 (30–65)	49 (32–64)	48 (33–63)	47 (33–61)	46 (32–59)	<0.001
**Gender**						<0.000
Male	2.809 (35.40%)	3.530 (43.40%)	4.260 (52.50%)	4.019 (62.30%)	1.166 (72.60%)	<0.001
Female	5.125 (64.60%)	4.613 (56.60%)	3.859 (47.50%)	2.537 (38.70%)	441 (27.40%)	
**Race**						<0.001
Mexican American	1.086 (13.69%)	1.341 (16.47%)	1.298 (15.99%)	1.052 (16.05%)	206 (12.82%)	
Other Hispanic	927 (11.68%)	855 (10.50%)	844 (10.40%)	603 (9.20%)	145 (9.02%)	
Non-Hispanic Blake	2.903 (36.59%)	3.279 (40.27%)	3.438 (42.35%)	2.939 (44.83%)	696 (43.31%)	
Non-Hispanic White	2.353 (29.66%)	1830 (22.47%)	1.562 (19.24%)	998 (15.22%)	277 (17.24%)	
Other Race	665 (8.38%)	838 (10.29%)	977 (12.03%)	964 (14.70%)	283 (17.61%)	
**Education level**						<0.001
Less than high school	2.550 (32.25%)	2.117 (26.07%)	1780 (21.97%)	1.249 (19.10%)	247 (15.40%)	
High school or equivalent	2.186 (27.65%)	2044 (25.17%)	1833 (22.62%)	1.300 (19.88%)	289 (18.02%)	
College or above	3.160 (39.96%)	3.951 (48.65%)	4.479 (55.28%)	3.986 (60.97%)	1.068 (66.58%)	
Not recorded	11 (0.14%)	9 (0.11%)	11 (0.14%)	3 (0.05%)	0 (0.00%)	
**Marital status**						
Living with partner	3.765 (50.41%)	4.498 (58.32%)	4.853 (62.40%)	4.082 (64.59%)	978 (63.14%)	
Living alone	3.604 (48.91%)	3.214 (41.68%)	2.924 (37.60%)	2.238 (35.41%)	571 (36.86%)	
FPIR	2.02 (0.86–2.90)	2.38 (1.07–3.70)	2.60 (1.16–4.25)	2.80 (1.26–4.84)	2.93 (1.29–5.00)	<0.001
BMI(kg/m^2^)	29.65 (24.40–33.50)	29.40 (24.30–33.00)	28.99 (24.00–32.40)	28.37 (23.70–31.70)	27.55 (23.40–30.50)	<0.001
Weight(kg)	80.46 (65.00–92.00)	81.41 (65.88–93.20)	81.64 (66.20–93.00)	81.86 (66.60–92.50)	81.22 (66.98–91.70)	0.039
**Smoking status**						<0.001
Yes	1724 (45.15%)	1.598 (41.09%)	1.546 (40.30%)	1.277 (41.62%)	323 (41.62%)	
No	2094 (54.85%)	2.291 (58.91%)	2.290 (59.70%)	1791 (58.38%)	453 (58.38%)	
**Alcoholic ≥ 4 drinks/day (%)**						0.292
Yes	508 (17.09%)	485 (15.66%)	502 (16.20%)	407 (16.20%)	121 (18.70%)	
No	2.464 (82.91%)	2.613 (84.34%)	2.597 (83.80%)	2.106 (83.80%)	526 (81.30%)	
**Physical activity**						<0.001
moderate activity/week	1.296 (33.10%)	1.617 (40.80%)	1710 (43.90%)	1.509 (48.50%)	445 (56.70%)	
others	2.619 (66.90%)	2.347 (59.20%)	2.186 (56.10%)	1.603 (51.50%)	340 (43.30%)	
**Sleep disorder**						0.038
Yes	1.090 (27.80%)	1.012 (25.50%)	985 (25.30%)	782 (25.10%)	178 (22.70%)	
No	2.828 (72.20%)	2.952 (74.50%)	2.910 (74.70%)	2.330 (74.80%)	607 (77.30%)	
**Essential hypertension**						<0.001
Yes	3.054 (38.49%)	2.936 (36.06%)	2.805 (34.55%)	2041 (31.13%)	481 (29.93%)	
No	4.880 (61.51%)	5.207 (63.94%)	5.314 (65.45%)	4.515 (68.87%)	1.126 (70.07%)	
**Diabetes**						<0.001
Yes	550 (14.36%)	542 (14.03%)	490 (12.92%)	326 (10.72%)	81 (10.09%)	
No	3.281 (85.64%)	3.321 (85.97%)	3.302 (87.08%)	2.714 (89.28%)	684 (89.41%)	
**Hypercholesterolemia**						0.04
Yes	2.447 (35.75%)	2.578 (36.52%)	2.699 (37.92%)	2.157 (38.17%)	480 (34.29%)	
No	4.398 (64.25%)	4.481 (63.48%)	4.418 (62.08%)	3.494 (61.83%)	920 (65.71%)	
**Cardiovascular health**						<0.001
Yes	640 (26.88%)	627 (25.20%)	625 (25.68%)	479 (24.93%)	121 (25.60%)	
No	1741 (73.12%)	1861 (74.80%)	1809 (74.32%)	1.442 (75.07%)	351 (74.40%)	
**Depression status**						<0.001
Yes	1.697 (27.30%)	1.670 (25.47%)	1.680 (25.30%)	1.370 (25.56%)	285 (21.56%)	
No	4.520 (72.70%)	4.887 (74.53%)	4.960 (74.70%)	3.990 (74.44%)	1.037 (78.44%)	

### 3.8 Subgroup analysis of copper intake with essential hypertension

**Figure 4:**
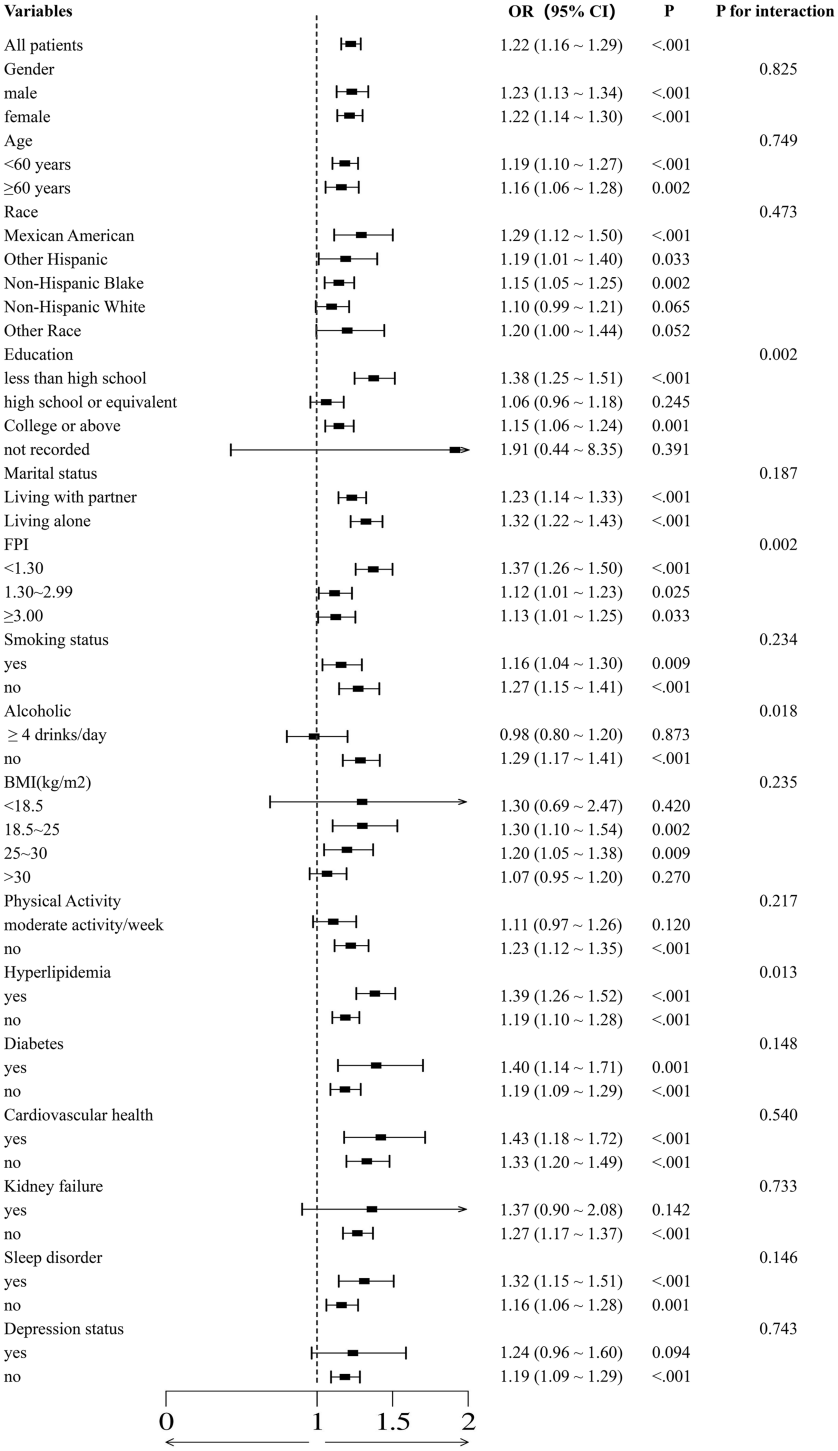
The association between normal and high copper intake and essential hypertension was evaluated using binomial logistic regression models, which were employed to calculate forest plots. The adjustments were made with reference to the covariates selected in the full binomial logistic regression model.

Figure 4 depicts the correlation between dietary copper intake and the prevalence of essential hypertension, accounting for various confounding factors. Across a wide range of subgroups defined by age, sex, race, marital status, smoking status, sleep patterns, cardiovascular disease, history of diabetes mellitus, and history of hyperlipidemia, higher dietary copper intake was consistently associated with an increased prevalence of essential hypertension, with *p*-values of less than 0.05 in each subgroup, indicating statistical significance. In stratified analyses, significant interactions were observed between dietary copper intake and factors such as smoking status, household income-to-poverty ratio, and the presence of hyperlipidemia with the risk of developing essential hypertension. The primary findings remained robust across several sensitivity analyses.

### 3.9 Binomial logistic regression analysis of copper intake with essential hypertension

As demonstrated in Table 2, in Model 1, which was not adjusted for any covariates, four high copper intake groups exhibited an elevated risk of developing essential hypertension in comparison to the normal copper intake group. Furthermore, all of these associations were statistically significant. The results indicated a statistically significant association between high copper intake and essential hypertension, with an odds ratio of 1.10 (*p* = 0.001, 95%CI:1.041–1.183), 1.186 (*p* < 0.001, 95% CI: 1.112–1.264), 1.384 (*p* < 0.001, 95%CI:1.292–1.483), and 1.465 (*p* < 0.001, 95%CI:1.305–1.645). After adjusting for sex, race, age, education level, and marital status, the risk of developing essential hypertension remained higher in the high copper intake group compared to the normal copper intake group (1.120, *p* = 0.003, 95% CI:1.040–1.206; 1.168, *p* < 0.001, 95%CI:1.084–1.259; 1.312, *p* < 0.001, 95%CI:1.211–1.422; and 1.292, *p* < 0.001, 95%CI:1.132–1.473). Model 3, which was adjusted for PIR, alcohol intake, smoking status, BMI, and physical activity, demonstrated statistical significance in all three groups, except for the high copper intake group, where the difference was 0.80–1.08 mg and was not statistically significant (*p* = 0.271, greater than 0). Model 4, which was adjusted for hypercholesterolemia, diabetes, cardiovascular disease, sleep disorders, and depressive state data, demonstrated a consistently higher incidence of essential hypertension in the high copper intake group compared to the normal copper intake group. The results were all statistically significant with odds ratios of 1.156 (*p* = 0.015, 95% CI: 1.029-1.298), 1.398 (*p*  < 0.001, 95% CI: 1.243-1.572), 1.496 (*p* < 0.001, 95% CI: 1.320-1.695) and 1.469 (*p* < 0.001, 95% CI. 95% CI: 1.196-1.804).

**Table 2:** The risk of essential hypertension was analyzed by multivariable-adjusted logistic regression for different levels of copper intake.

Variable	Model 1		Model 2		Model 3		Model 4	
Hypertension	OR (95% CIs)	*p*-value	OR (95% CIs)	*p*-value	OR (95% CIs)	*p*-value	OR (95% CIs)	*p*-value
Copper intake								
<0.80 mg	1		1		1		1	
0.80–1.08 mg	1.110 (1.041–1.183)	0.001	1.120 (1.040–1.206)	0.003	1.063 (0.954–1.184)	0.271	1.156 (1.029–1.298)	0.015
1.08–1.45 mg	1.186 (1.112–1.264)	<0.001	1.168 (1.084–1.259)	<0.001	1.231 (1.103–1.374)	<0.001	1.398 (1.243–1.572)	<0.001
1.45–2.29 mg	1.384 (1.292–1.483)	<0.001	1.312 (1.211–1.422)	<0.001	1.317 (1.172–1.480)	<0.001	1.496 (1.320–1.695)	<0.001
>2.29 mg	1.465 (1.305–1.645)	<0.001	1.292 (1.132–1.473)	<0.001	1.237 (1.025–1.491)	0.026	1.469 (1.196–1.804)	<0.001

### 3.10 Restricted cubic spline of copper intake with essential hypertension

**Figure 5:**
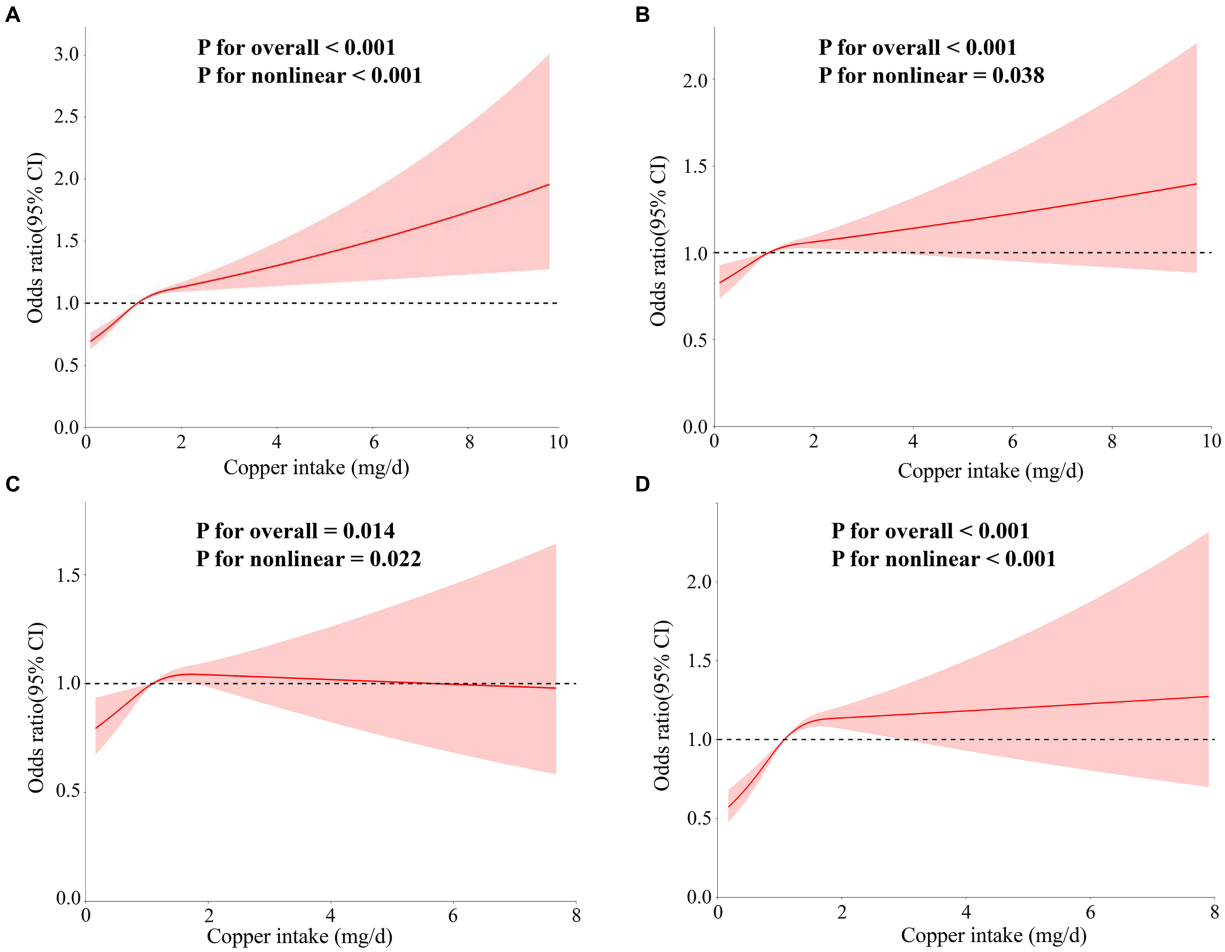
The correlation between copper intake and essential hypertension was assessed using RCS curves. The models were as follows: **(A)** Model 1 without adjustment for covariates; **(B)** Model 2 adjusted for age, sex, race, education, and marital status variables; **(C)** Model 3 adjusted for PIR, alcohol intake, smoking status, body mass index (BMI), and physical activity variables; **(D)** models adjusted for hypercholesterolemia, diabetes, cardiovascular disease, renal failure, and sleep disorder variables.

As shown in Figure 5, analyses of the RCS without adjustment for covariates revealed an “L”-shaped nonlinear relationship between hypertension and copper intake (*p* for overall <0.001, *p* for nonlinear <0.001). In models 2 and 4, the “L”-shaped relationship was still present (*p* for overall <0.001, *p* for nonlinear = 0.038; *p* for overall <0.001, *p* for nonlinear <0.001), whereas a “U”-shaped relationship was shown in model 3 (*p* for overall = 0.014, *p* for nonlinear = 0.022).

## 4 Discussion

After screening 15 micronutrients and removing weak instrumental variables, a two-sample Mendelian randomization analysis identified copper and potassium as being associated with essential hypertension. Sensitivity analyses further supported these findings (27). To account for potential interactions between trace elements, a multivariate Mendelian randomization analysis was conducted, which revealed that potassium was not associated with essential hypertension, while copper remained a significant risk factor. To validate these results, a logistic regression analysis using data from the NHANES database was performed. The findings confirmed that copper intake is associated with the prevalence of essential hypertension, consistent with the results obtained from Mendelian randomization.

The study data revealed that women and individuals with sleep disorders represented a larger proportion of those with low dietary copper intake. This finding may be explained by the unique physiological characteristics of women, such as cyclical blood loss, which can lead to copper depletion, and specific conditions like pregnancy and lactation, where increased copper requirements may result in insufficient intake if not adequately supplemented through diet or additional sources. Additionally, certain dietary habits or restrictions, such as vegetarianism, can further impact copper intake. The observed correlation between sleep disorders and low copper intake may be due to copper deficiency’s direct or indirect effects on the brain’s regulatory processes of excitation and inhibition, potentially leading to disrupted sleep and insomnia (28). Moreover, research has shown that sleep fragmentation can exacerbate myocardial ischemia–reperfusion injury by promoting copper overload in cardiomyocytes (29).

In clinical studies, Karolina Kedzierska et al. demonstrated a negative correlation between Na+/K+/Cl\—cotransport activity and plasma copper concentration in hypertensive patients (Rs = −0.579, *p* < 0.05). Similarly, ex-Na+/Li + activity was also negatively correlated with plasma copper concentration (Rs = −0.508, *p* < 0.05). These findings suggest that plasma copper concentration may increase the risk of essential hypertension by affecting sodium transport activity in the erythrocyte membrane (30). Using data from the NHANES database, Liu et al. observed a nonlinear relationship between serum copper concentration and elevated blood pressure in U. S. children and adolescents, finding that higher serum copper levels were significantly associated with hypertension in this population (31). Furthermore, a study found that the logarithmic transformation of dietary copper intake in hypertensive patients was significantly associated with longer telomere length, indicating that copper may contribute to the development and progression of essential hypertension by influencing telomere length (32). In another study, Pan He et al. identified a U-shaped relationship between dietary copper intake and new-onset hypertension, with an inflection point at approximately 1.57 mg/day among Chinese adults (14). The difference in our findings may be attributed to the use of different databases and the study populations, as Pan He’s research focused on a Chinese cohort, whereas our study analyzed a U. S. population. Several other clinical studies have also explored the relationship between copper and cardiovascular diseases, including hypertension. For example, Muñoz-Bravo et al. found that elevated serum copper levels were associated with an increased risk of cardiovascular events (33). Zhou et al. identified blood copper as a novel risk factor for subclinical carotid atherosclerosis and demonstrated that mixtures of copper, cadmium, and lead may exert a synergistic pro-atherosclerotic effect (34). Additionally, Zhang et al. reported that baseline plasma copper levels were significantly and positively associated with the risk of first stroke in Chinese hypertensive patients (35).

Experimental animal studies suggest that copper levels may directly regulate blood pressure by influencing angiotensin, the nervous system, or kidney function. For instance, in angiotensin II (Ang II)-induced hypertension, the antioxidant 1 copper chaperone (Atox1) raises blood pressure by reducing extracellular oxygen anions and increasing the expression and activity of vascular superoxide dismutase 3 (SOD3) (36). Additionally, angiotensin-converting enzyme II (ACE2) significantly reduces copper levels in the vasculature of Atox1-deficient rats, likely due to an Ang II-induced increase in circulating SOD3, which aligns with the observed reduction in copper levels in tissues such as the liver and kidneys of hypertensive rodents (36). It has been demonstrated that ATP7A, a copper-transporting protein, plays a critical role in Ang II-induced hypertension and the regulation of endothelial function by modulating SOD3 activity and vascular superoxide anion production (37). Conversely, in norepinephrine-induced hypertension, the generation of vascular oxygen anions was unaffected in ATP7A mutant mice with impaired copper transport, a finding consistent with observations in SOD3 knockout mice (38). Furthermore, the basal and Ang II-induced increases in vascular SOD3-specific activity were significantly inhibited in ATP7A mutant mice. In cultured vascular smooth muscle cells and mouse aorta, Ang II stimulation promoted the binding of ATP7A to SOD3, potentially enhancing SOD3-specific activity by facilitating copper delivery from ATP7A to SOD3. Copper transport proteins in the kidney and central nervous system also play roles in blood pressure regulation. Atox1 expression has been observed in several brain regions, including the choroid plexus, which is part of the circumventricular organs (CVOs), and ATP7A is also expressed in the choroid plexus (39). Deficiency of SOD3 in the CVO has been reported to elevate both basal and post-Ang II injection blood pressure, partly by modulating sympathetic outflow (40). It is speculated that brain-expressed Atox1 may regulate blood pressure by modulating SOD3 activity or its copper chaperone function, potentially affecting other secreted copper enzymes (41). Additionally, SOD3 gene transfer has been shown to reduce renal sodium retention in hypertensive rats (42). Therefore, Atox1 and ATP7A likely play significant roles in hypertension by regulating both kidney and brain functions.

Multiple lines of evidence support the role of vascular copper transporter proteins in regulating both the activity of copper-dependent enzymes, such as SOD1 and SOD3, and the overall intracellular copper content. For instance, Clegg MS et al. found that copper levels were altered in the tissues of hypertensive rats compared to healthy controls, with the degree of alteration correlating with the severity of hypertension (43). While the current study suggests that copper may have an interventional effect on hypertension, the precise mechanisms remain unclear. This study is notable as the first cross-sectional analysis to utilize the NHANES database, encompassing a 12 year cohort of over 50,000 subjects, to identify copper intake as a risk factor for essential hypertension. By combining Mendelian randomization with NHANES data, we were able to assess causality, thereby overcoming the limitations of traditional observational studies, which often struggle to distinguish between causality and correlation. This approach enhances causal inference (44, 45). The National Health and Nutrition Examination Survey (NHANES) is a comprehensive database offering a vast array of epidemiological data, which significantly bolsters the statistical reliability of our findings. This study confirms a dose–response relationship between copper intake and the risk of essential hypertension. The results can inform the development of targeted hypertension prevention strategies and dietary interventions in high-risk populations, as well as provide a foundation for future laboratory studies aimed at elucidating the underlying biological mechanisms.

Our study has several limitations. Individual differences in food selection, digestive processes, and absorptive capacity may influence actual copper intake, introducing variability that is difficult to control. Additionally, the database lacks renal ultrasound, CT, or MRI assessments of renal structure and function, as well as imaging of the adrenal glands, which could help rule out conditions such as adrenal adenomas or hyperplasia. This absence of detailed diagnostic testing limits our ability to accurately diagnose conditions like primary aldosteronism and Cushing’s syndrome, leading to some imprecision in distinguishing primary hypertension from secondary forms. However, given the low prevalence of secondary hypertension in the hypertensive population, our study remains feasible and relevant. Another limitation involves the assumptions inherent in Mendelian randomization (MR). MR presumes that gene effects are consistent across all individuals, but in reality, gene effects may vary depending on environmental factors or other contextual influences. Such gene-environment interactions could complicate MR results, potentially affecting the accuracy of causal inferences (46).

## Data Availability

The original contributions presented in the study are included in the article/Supplementary material, further inquiries can be directed to the corresponding authors.

## Footnotes

^1^ https://www.ebi.ac.uk/gwas

^2^ https://www.ncbi.nlm.nih.gov/pubmed

^3^ https://r10.finngen.fi/

^4^ https://risteys.finregistry.fi/endpoints/I9_HYPTENSESS

^5^ https://www.cdc.gov/nchs/nhanes/index.Htm
