# Supplementary material for: Association between copper intake and essential hypertension: dual evidence from Mendelian randomization analysis and the NHANES database
Source: Front Nutr. 2024 Aug 29;11:1454669. doi: 10.3389/fnut.2024.1454669 (PMC11391934; doi:10.3389/fnut.2024.1454669)
Supplement: Supplementary file 1 [file Table_1.docx]

**STROBE-MR checklist of recommended items to address in reports of Mendelian randomization studies**^1^ ^2^

| **Item No.** | **Section** | **Checklist item** | **Page No.** | **Relevant text from manuscript** |
| --- | --- | --- | --- | --- |
| 1 | **TITLE and ABSTRACT** | Indicate Mendelian randomization (MR) as the study’s design in the title and/or the abstract if that is a main purpose of the study | 1 | The aim of this study was to assess the potential causal relationship between 15 major trace elements and FM risk using Mendelian randomisation and to explore the specific role of significantly associated trace elements in an essential hypertension. |
|  | **INTRODUCTION** |  | 1 |  |
| 2 | **Background** | Explain the scientific background and rationale for the reported study. What is the exposure? Is a potential causal relationship between exposure and outcome plausible? Justify why MR is a helpful method to address the study question | 1 | In recent years, it has been found that trace elements in the human body are associated with the development of a variety of diseases, including essential hypertension. For example, selenium, as an important antioxidant, is involved in the protection of the cardiovascular system from oxidative stress damage and may help prevent cardiovascular disease. Zinc, on the other hand, may be involved in blood pressure regulation by influencing immune function and angiotensin-converting enzyme activity. Organic germanium has been observed to have a sustained hypotensive effect, effectively lowering systolic and diastolic blood pressure and improving hypertension symptoms. Copper, on the other hand, has been implicated in blood pressure regulation and affects blood pressure by influencing the synthesis of norepinephrine.In addition, several other trace elements have been found to be associated with essential hypertension. |
| 3 | **Objectives** | State specific objectives clearly, including pre-specified causal hypotheses (if any). State that MR is a method that, under specific assumptions, intends to estimate causal effects | 1 | In our study, exposure to 15 micronutrients resulted in primary hypertension as the outcome indicator. As for primary hypertension, there is evidence to suggest that abnormal micronutrient metabolism may be related to disease progression. The second paragraph of the manuscript discusses many studies on the association between trace elements and primary hypertension. Therefore, MR is used to investigate whether the above exposure is related to primary hypertension, with the ultimate goal of preventing or delaying disease progression. |
|  | **METHODS** |  |  |  |
| 4 | **Study design and data sources** | Present key elements of the study design early in the article. Consider including a table listing sources of data for all phases of the study. For each data source contributing to the analysis, describe the following: |  | The GWAS data for the 15 trace elements in our study data sources were obtained from IEU OpenGWAS.The specific ID numbers are shown in Supplementary Table 1, and the GWAS data for FM were obtained from the most recent R10 version of the FinnGen database, with the ID number finngen_R10_I9_HYPTENSESS.The information on the data is presented in Supplementary Table 1. |
|  | a) | Setting: Describe the study design and the underlying population, if possible. Describe the setting, locations, and relevant dates, including periods of recruitment, exposure, follow-up, and data collection, when available. |  | Information about the ieu database and the FinnGen biobank is described in the data sources, including when the samples were collected (see Supplementary Table 1 for details), the study site (Europe), and the study population (European origin). |
|  | b) | Participants: Give the eligibility criteria, and the sources and methods of selection of participants. Report the sample size, and whether any power or sample size calculations were carried out prior to the main analysis |  | This study reports the sample size of study subjects 10336 and 2,603 cases, respectively. The outcome data included 102864 essential hypertension patients and 289117 controls. The method of calculating sample size was not reported. |
|  | c) | Describe measurement, quality control and selection of genetic variants |  | Instrumental variables in the study were obtained from the IEU database and the FinnGen biobank, and the screening process and criteria for the genetic variant instrumental variables are also reported in the article, as described in the methodology section of the article. |
|  | d) | For each exposure, outcome, and other relevant variables, describe methods of assessment and diagnostic criteria for diseases |  | This study describes the definition of the outcome in the selection of genetic instrumental variables for methodology and provides the references on which the definition is based. The diagnosis of essential hypertensive disease requires a systolic blood pressure ≥140 mmHg and/or a diastolic blood pressure ≥90 mmHg measured on three non-simultaneous occasions on the same day without the use of antihypertensive medication. |
|  | e) | Provide details of ethics committee approval and participant informed consent, if relevant |  | Ethical approval and informed consent of the patients were obtained during the collection of gaws data for this study and the current MR was also approved by the Swedish Ethical Review Board, approval number: 2019-02793 |
| 5 | **Assumptions** | Explicitly state the three core IV assumptions for the main analysis (relevance, independence and exclusion restriction) as well assumptions for any additional or sensitivity analysis |  | This paper provides a preliminary description of the three core hypotheses, but the core hypotheses are reflected in the analytical methods. In addition, three methods for sensitivity analyses and horizontal polytropy detection are described: the weighted median method, MR-Egger, and MR-PRESSO, and the use of the F-statistic to calculate statistical validity and the Q-statistic to detect heterogeneity is reported. |
| 6 | **Statistical methods: main analysis** | Describe statistical methods and statistics used |  |  |
|  | a) | Describe how quantitative variables were handled in the analyses (i.e., scale, units, model) |  | Studies reported analyses using random-effects models when there were >3 instrumental variables for genetic variants, otherwise fixed-effects models were used; statistical effect sizes or units of measurement for exposure, outcome, and relevant covariates were not transformed and therefore not reported |
|  | b) | Describe how genetic variants were handled in the analyses and, if applicable, how their weights were selected |  | Strict criteria are used to determine tool variables for alternative exposure, including limiting p values and excluding linkage disequilibrium. This method has been widely used in previous MR research. |
|  | c) | Describe the MR estimator (e.g. two-stage least squares, Wald ratio) and related statistics. Detail the included covariates and, in case of two-sample MR, whether the same covariate set was used for adjustment in the two samples |  | The genetic tools used to report exposure in this article were derived from the results of GWAS, and the study population did not use genetic modeling to adjust for age, gender, or study location analysis. In addition, the article also provides a detailed introduction to specific MR statistical methods and software used. |
|  | d) | Explain how missing data were addressed |  | No lost data was found. |
|  | e) | If applicable, indicate how multiple testing was addressed |  | Although only copper and Potassium are meaningful results in two simple samples, in order to eliminate possible confounding factors, only copper is meaningful after multivariate analysis. |
| 7 | **Assessment of assumptions** | Describe any methods or prior knowledge used to assess the assumptions or justify their validity |  | TheF-statisticwasusedtoestimatestatisticalpowerinthisstudyandnoothermethodwasused |
| 8 | **Sensitivity analyses and additional analyses** | Describe any sensitivity analyses or additional analyses performed (e.g. comparison of effect estimates from different approaches, independent replication, bias analytic techniques, validation of instruments, simulations) |  | In this study, Cochran Q was used to test for heterogeneity; if P>0.05, it indicates that there is no heterogeneity, and vice versa. If the instrumental variables can directly affect the outcome variables without the exposure factor then it indicates the presence of horizontal pleiotropy, which violates the exclusivity assumption of MR. If horizontal polyvalence exists in the results of MR analyses it indicates that they are not reliable |
| 9 | **Software and pre-registration** |  |  |  |
|  | a) | Name statistical software and package(s), including version and settings used |  | TwoSampleMR package (version 0.5.6) in R 4.3.2. |
|  | b) | State whether the study protocol and details were pre-registered (as well as when and where) |  | Unfortunately, the study protocol and details were not pre-registered. At the initiation of the study, we did not initially consider or recognize the necessity of pre-registration. We appreciate your understanding of this limitation. |
|  | **RESULTS** |  |  |  |
| 10 | **Descriptive data** |  |  |  |
|  | a) | Report the numbers of individuals at each stage of included studies and reasons for exclusion. Consider use of a flow diagram |  | The example text provides the number of sample populations in the methodology section and does not provide information about the populations in the results. Information on excluded populations with reasons for exclusion is not provided and the flow chart is shown in Figure 1 (A). |
|  | b) | Report summary statistics for phenotypic exposure(s), outcome(s), and other relevant variables (e.g. means, SDs, proportions) |  | Please refer to the Supplementary Table 2 for summary statistical data on phenotype exposure, outcomes, and other related variables (such as mean, SD, proportion) reported. |
|  | c) | If the data sources include meta-analyses of previous studies, provide the assessments of heterogeneity across these studies |  | No data from meta-analysis found |
|  | d) | For two-sample MR:  i.  Provide justification of the similarity of the genetic variant-exposure associations between the exposure and outcome samples  ii.  Provide information on the number of individuals who overlap between the exposure and outcome studies |  | This study is a two-sample and multivariate MR, and in Supplementary Table 1 all sample populations are listed as being from Europe, so there is very little ethnographic heterogeneity, and adjustments were made for age, gender, and other factors. The study did not report information on overlapping populations. |
| 11 | **Main results** |  |  |  |
|  | a) | Report the associations between genetic variant and exposure, and between genetic variant and outcome, preferably on an interpretable scale |  | This study reports the content of the entry in Supplementary Table 2, including the number of instrumental variables SNPs, sample size, adjusted confounding factors, correlation between exposure instrumental variables, and statistical validity. |
|  | b) | Report MR estimates of the relationship between exposure and outcome, and the measures of uncertainty from the MR analysis, on an interpretable scale, such as odds ratio or relative risk per SD difference |  | The majority of this study's space in the results is devoted to the reporting of the content of the entry, e.g. the OR for the association of copper with was essential hypertension 1.025 with a 95% CI (1.008, 1.041) |
|  | c) | If relevant, consider translating estimates of relative risk into absolute risk for a meaningful time period |  | In the discussion section, it is pointed out that there is a significant correlation between the logarithmic conversion of dietary copper intake and the increase of telomere length in patients with hypertension, which suggests that copper may participate in the occurrence and development of hypertension by changing telomere length. |
|  | d) | Consider plots to visualize results (e.g. forest plot, scatterplot of associations between genetic variants and outcome versus between genetic variants and exposure) |  | Figure 2 and Figure 3 |
| 12 | **Assessment of assumptions** |  |  |  |
|  | a) | Report the assessment of the validity of the assumptions |  | The statistical validity of the tool variables in each association is reported in the methodology, and the results are represented by F statistics. Q statistics are used to test the heterogeneity of the statistical model and evaluate its stability. |
|  | b) | Report any additional statistics (e.g., assessments of heterogeneity across genetic variants, such as *I^2^*, Q statistic or E-value) |  | The example text reports the results of the Q statistic in the results, demonstrating its significance. |
| 13 | **Sensitivity analyses and additional analyses** |  |  |  |
|  | a) | Report any sensitivity analyses to assess the robustness of the main results to violations of the assumptions |  | This example article calculates the results of five kinds of MR analysis, but only provides the MR analysis results obtained by two analysis methods, including the weighted median method and the IVW method, as shown in figure 2. In addition, the paper also shows the sensitivity analysis, including MREgger and MRPleiotropy methods. |
|  | b) | Report results from other sensitivity analyses or additional analyses |  | The sample text reports the results of other analyses, such as the Leave-One-out method in Figure 3 (B). |
|  | c) | Report any assessment of direction of causal relationship (e.g., bidirectional MR) |  | This study has controlled for confounding under multivariate Mendelian randomisation analyses and did not report reverse results. |
|  | d) | When relevant, report and compare with estimates from non-MR analyses |  | Non-MR results are not reported in the Results section of this paper at this time, but are discussed in the Discussion section . |
|  | e) | Consider additional plots to visualize results (e.g., leave-one-out analyses) |  | Figure 3 |
|  | **DISCUSSION** |  |  |  |
| 14 | **Key results** | Summarize key results with reference to study objectives |  | The sample text reports all the key findings in the first paragraph of the discussion: trace element copper is a risk factor for essential hypertension |
| 15 | **Limitations** | Discuss limitations of the study, taking into account the validity of the IV assumptions, other sources of potential bias, and imprecision. Discuss both direction and magnitude of any potential bias and any efforts to address them |  | The limitations of the study are discussed at great length in the example essay in terms of the data sources, the three main hypotheses and the analytical process in a detailed manner. |
| 16 | **Interpretation** |  |  |  |
|  | a) | Meaning: Give a cautious overall interpretation of results in the context of their limitations and in comparison with other studies |  | This entry is discussed at maximum length in the example paper, and the MR results are rationalised by comparison with several published studies. |
|  | b) | Mechanism: Discuss underlying biological mechanisms that could drive a potential causal relationship between the investigated exposure and the outcome, and whether the gene-environment equivalence assumption is reasonable. Use causal language carefully, clarifying that IV estimates may provide causal effects only under certain assumptions |  | In the discussion, it is mainly considered that the trace element copper may affect essential hypertension through various potential biological mechanisms. |
|  | c) | Clinical relevance: Discuss whether the results have clinical or public policy relevance, and to what extent they inform effect sizes of possible interventions |  | The instrumental variable SNP in this paper may be subject to a number of other possible confounding influences, and there may also be interactions between genetic and environmental factors, so the MR results are also subject to uncertainty, but conclusions can still be drawn.The discussion results of this paper can provide some reference value for the standard setting of dietary intake, especially for people with family history of hypertension. |
| 17 | **Generalizability** | Discuss the generalizability of the study results (a) to other populations, (b) across other exposure periods/timings, and (c) across other levels of exposure |  | This study also combined with the NHANES cross-sectional study in the United States, which greatly reduced the problem of low population applicability. |
|  | **OTHER INFORMATION** |  |  |  |
| 18 | **Funding** | Describe sources of funding and the role of funders in the present study and, if applicable, sources of funding for the databases and original study or studies on which the present study is based |  | The authors declare that financial support was received for the research, authorship, and/or publication of this article. The authors disclose that they have no business or financial associations that could create potential conflicts of interest. This work was financially supported by the First Affiliated Hospital of Guangzhou University of Traditional Chinese Medicine 2024 Hospital Young and Middle-aged Key Talent Cultivation Project [grant numbers A1-2601-24-414-110Z77]. |
| 19 | **Data and data sharing** | Provide the data used to perform all analyses or report where and how the data can be accessed, and reference these sources in the article. Provide the statistical code needed to reproduce the results in the article, or report whether the code is publicly accessible and if so, where |  | The GWAS data for the 15 micronutrients were sourced from the IEU OpenGWAS database, with specific ID numbers listed in Supplementary Table 1. Additionally, the GWAS data for fibromyalgia (FM) were obtained from the latest R10 version of the FinnGen database, identified by the ID number finngen_R10_I9_HYPTENSESS. |
| 20 | **Conflicts of Interest** | All authors should declare all potential conflicts of interest |  | The authors declare that this study was conducted without any commercial or financial relationship, which could be interpreted as a potential conflict of interest. |

This checklist is copyrighted by the Equator Network under the Creative Commons Attribution 3.0 Unported (CC BY 3.0) license.

1. Skrivankova VW, Richmond RC, Woolf BAR, Yarmolinsky J, Davies NM, Swanson SA, et al. Strengthening the Reporting of Observational Studies in Epidemiology using Mendelian Randomization (STROBE-MR) Statement. JAMA. 2021;under review.

2. Skrivankova VW, Richmond RC, Woolf BAR, Davies NM, Swanson SA, VanderWeele TJ, et al. Strengthening the Reporting of Observational Studies in Epidemiology using Mendelian Randomisation (STROBE-MR): Explanation and Elaboration. BMJ. 2021;375:n2233.
